# S_TransNeXtM: a pig behavior recognition model based on the TransNeXtM and the sLSTM

**DOI:** 10.3389/fvets.2025.1674842

**Published:** 2025-10-02

**Authors:** Wangli Hao, Xinyuan Hu, Yakui Xue, Hao Shu, Meng Han

**Affiliations:** ^1^College of Software, Shanxi Agricultural University, Jinzhong, Shanxi, China; ^2^School of Information Science and Engineering, Shanxi Agricultural University, Jinzhong, Shanxi, China; ^3^Hangzhou Dianzi University, Hangzhou, Zhejiang, China

**Keywords:** S_TransNeXtM, TransNeXtM, temporal sequence, pig behavior recognition, sLSTM

## Abstract

Pig behavior recognition serves as a crucial indicator for monitoring health and environmental conditions. However, conventional pig behavior recognition methods are limited in their ability to effectively extract image features and analyze long sequence dependencies, ultimately reducing pig behavior recognition performance. To address these challenges, we proposes a pig behavior recognition model S_TransNeXtM which leverages both spatial and temporal information underlying the video. Specifically, an innovative backbone, named TransNeXtM, has been developed for the spatial domain. It incorporates a bio-inspired Aggregated Attention Mechanism, a Convolutional GLU, and a Mamba unit, which allows the model to capture more discriminative global and local features. For the temporal domain, the sLSTM is proposed to process sequence data by utilizing an exponential gating mechanism and a stabilizer state. This design allows the model to establish longer temporal sequence dependencies, outperforming conventional GRU and LSTM. Based on the above insights, the S_TransNeXtM enhances the performance of pig behavior recognition. Experimental results demonstrate that the proposed S_TransNeXtM model achieves the state-of-the-art performance in pig behavior recognition task. Consequently, the S_TransNeXtM attains an accuracy of 94.53%, marking an improvement of up to 11.32% over previous benchmarks.

## Highlights

- Conventional pig behavior recognition methods are limited in their ability to effectively extract image features and analyze long sequence dependencies, ultimately reducing pig behavior recognition performance.
- A S_TransNeXtM model for precise pig behavior recognition, by jointly optimizing both spatial and temporal domains.
- We first develop a new TransNeXtM module to deal with the spatial features underlying pig behavior videos.
- We ingeniously employ the sLSTM to investigate the temporal features of the corresponding pig behavior videos.

## 1 Introduction

Pig behavior recognition plays an important role in feeding management (1). Through accurate identification of pig behavior, we can understand its physiological and psychological needs, and then targeted adjustment of the feeding environment and feeding formula, improve production efficiency (2). Therefore, pig behavior recognition is not only a key link in improving the level of feeding management, but also an important means of promoting the sustainable development of animal husbandry.

Traditional pig behavior recognition methods mainly rely on manual observation and sensor technology (3). While manual observation is intuitive, it is subjective, time-consuming, and limits the depth of exploration into pigs' physiological and psychological needs (4, 5). Sensor technology, using speed, infrared, and sound sensors, accurately captures pig movement for precise behavior identification and monitoring. However, sensor layout is complex and prone to detachment, potentially affecting data accuracy and continuity, and posing risks to pig growth and health.

Therefore, with the progress of science and technology, more and more researchers began to explore the use of computer vision, deep learning and other advanced technologies for pig behavior recognition (6, 7). These methods improve the accuracy and efficiency of identification, and provide strong technical support for the optimization of feeding management.

Hengyi et al. (8) pioneeringly integrated the Temporal Shift Module (TSM) into various mainstream 2D convolutional neural network architectures, including ResNet50, ResNeXt50, DenseNet201, and ConvNeXt-t. This innovation significantly enhanced the model's capability to recognize pig aggression behaviors, achieving an impressive accuracy rate of 95.69% in experiments.

Yue et al. (9) focused on behavior analysis within video sequences by introducing a hybrid model that integrates Convolutional Neural Networks (CNNs) with Gated Recurrent Units (GRUs). This model effectively leverages the spatial feature extraction capabilities of CNNs and the temporal sequence processing strengths of GRUs, achieving an accuracy rate of 94.8% in experiments.

Lili et al. (10) delved into pig expression recognition, integrating ASPP and CReToNeXt modules into the ASP-YOLOv5 model. This innovation optimized feature extraction and fusion processes, resulting in a mean Average Precision (mAP) of 93.2%.

Junjie et al. (11) focused on classifying interactive behaviors among pigs by designing a deep learning framework that fused Convolutional Neural Networks (CNNs) with Long Short-Term Memory (LSTM). Under fixed training set sizes, the framework was validated through multiple methods, including random validation, temporal blocking validation, and feeder blocking validation, achieving an average accuracy of 96.8%.

Ma et al. (12) achieved remarkable results in model optimization, proposing the optimized M-YOLOv4-C network model. This model adopted the lightweight MobileNet-v3 as its core architecture and incorporated depthwise separable convolution into YOLOv4's feature extraction network. This innovation not only boosted the model's accuracy to 98.15% but also achieved a detection speed of 106.3 frames per second while keeping the model size at 44.74 MB, enabling real-time applications for pig behavior recognition.

Zhang et al. (13) proposed a Transformer-based Neural Network (TNN) model that leveraged attention heatmap visualization techniques to precisely pinpoint and analyze critical image regions, demonstrating exceptional performance in piglet behavior recognition. Notably, even with reduced parameter counts and computational complexity, the TNN model maintained outstanding recognition efficacy.

Despite some progress in deep learning for pig behavior recognition, challenges remain in accurately extracting image features and simultaneously analyzing long temporal sequence data.

The key contributions of this paper are 3 fold:

- This paper introduces a S_TransNeXtM model for precise pig behavior recognition, by jointly optimizing both spatial and temporal domains. Specifically, it leverages TransNeXtM to capture more abundant global and local discriminative features in the spatial domain, facilitating the discrimination of subtle pig behavioral differences. Additionally, sLSTM (spatial Long Short-Term Memory) is creatively employed in the temporal domain to handle long sequence dependencies.
- We first develop a new TransNeXtM module to deal with the spatial features underlying pig behavior videos. Concretely, the module incorporates the Aggregated Attention Mechanism and Convolutional GLU (Convolutional Gate-controlled Linear Unit) for capturing global and local features, respectively. To further enhance its discriminative capability, the Mamba unit is innovatively introduced into the module. This design promotes the filtering of noise and redundant information that may interfere with discrimination. Consequently, it enable the model to capture subtle differences in pig behavior, thereby significantly improves the performance of the model.
- In this paper, we ingeniously employ the sLSTM to investigate the temporal features of the corresponding pig behavior videos. This module can effectively capture the long temporal dependencies through exponential gating and stabilized states. Thus, a notable enhancement of the model's performance can be obtained.

## 2 Materials and methods

### 2.1 Dataset

**Figure 1:**
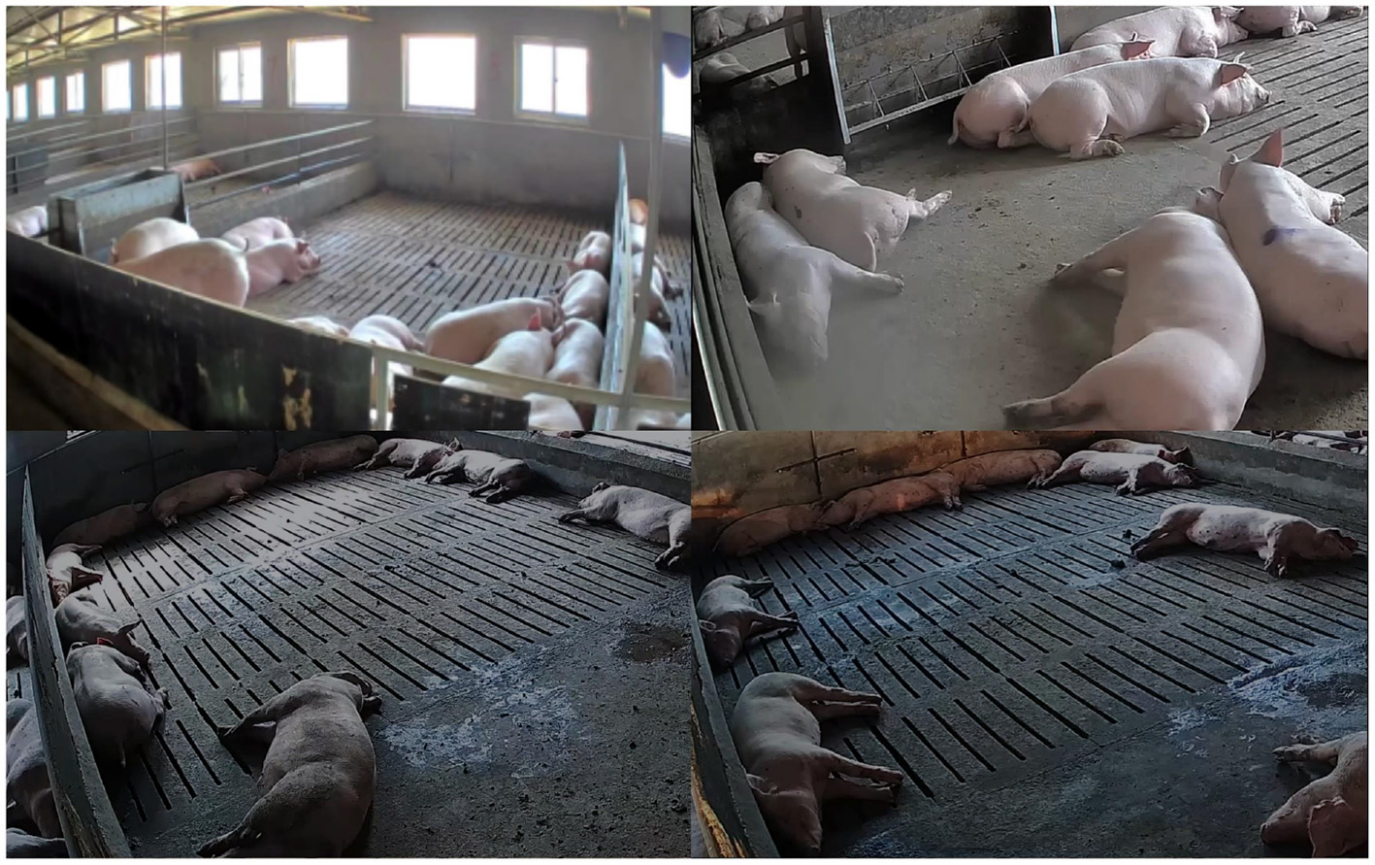
View of the camera inside some pig houses.

The pig behavior recognition data is collected from the pig breeding base of Nonglvyuan Agriculture Co., LTD., Xiangfen County, Linfen City, Shanxi Province. Specifically, the data collection range from August 12, 2022 to September 25, 2022. The farm comprises six pig houses, each housing 6-month-old ternary breed pigs, with an average of 10 pigs per house. Concretely, cameras have been installed in all six pig houses, employing the Hikvision DS-2DE3Q120MY-T/GLSE. These cameras are mounted at a 45-degree angle to the side of each pig house, 3 meters above the ground. They are capable of capturing RGB color space video with a resolution of 1,920*1,080 pixels and a sampling rate of 25 frames per second. A visual depiction of the camera's view inside one of the pig houses is presented in the Figure 1.

**Figure 2:**
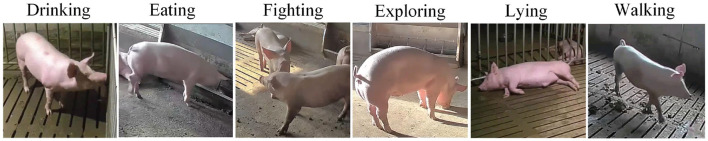
Examples of six pig behavior categories.

Finally, after data collection, we obtain 1.5 TB of pig video data, which covers more than 5,000 video files. Subsequently, video clips with durations ranging from 5 to 10 s are selected. After this video preprocessing, we identify six pig behavior categories: Drinking, Eating, Fighting, Exploring, Lying, and Walking, as shown in Figure 2. Specifically, Drinking was characterized by oral suction at water sources. Eating by head-in-trough mastication. Fighting by aggressive pushing or biting for resource competition. Exploring constitutes sustained active contact exceeding 3 s with environmental elements (14). Lying by resting postures with extended or curled limbs. Walking by limb-alternation displacement for spatial movement (15).

Each category comprised approximately 450 videos, with the total dataset containing 2,755 videos. Each video consists of 24 image frames, which are arranged in temporal order to form a complete video segment. The inter-frame difference method was used to detect blurry frames, and a total of 3.7% of low-quality segments in the original data were eliminated. Within each category, the training and test sets were partitioned in an 8:2 ratio. Further, the detailed statistics of the number of each pig behavior are presented in Table 1.

**Table 1:** Statistical of the number of videos for different categories in the dataset.

**Behavior category**	**Drinking**	**Eating**	**Fighting**	**Exploring**	**Lying**	**Walking**
Number	426	489	438	485	474	443
Training	341	391	350	388	379	354
Test	85	98	88	97	95	89

### 2.2 S_TransNeXtM

**Figure 3:**
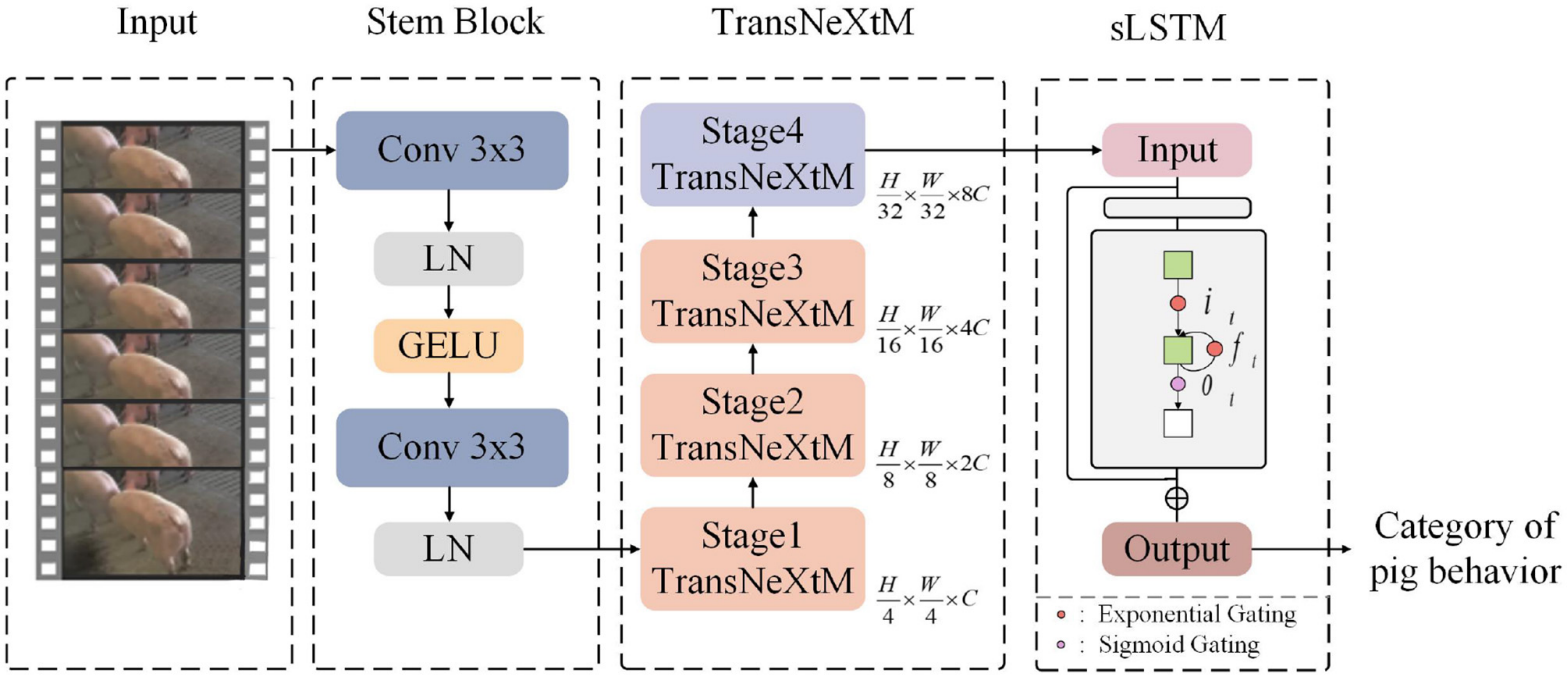
The pipeline of the S_TransNeXtM.

In order to improve the performance of pig behavior recognition, this paper proposes a novel model, S_TransNeXtM, which leverages both spatial and temporal information. Specifically, the S_TransNeXtM model which comprised of two modules: the TransNeXtM and the sLSTM(spatial Long Short-Term Memory) respectively, as shown in Figure 3. Following, we will elaborate on these two modules in detail.

#### 2.2.1 TransNeXtM module

This subsection introduces a novel pig behavior recognition backbone module, named TransNeXtM, aiming to capture spatial information in the corresponding task.

**Figure 4:**
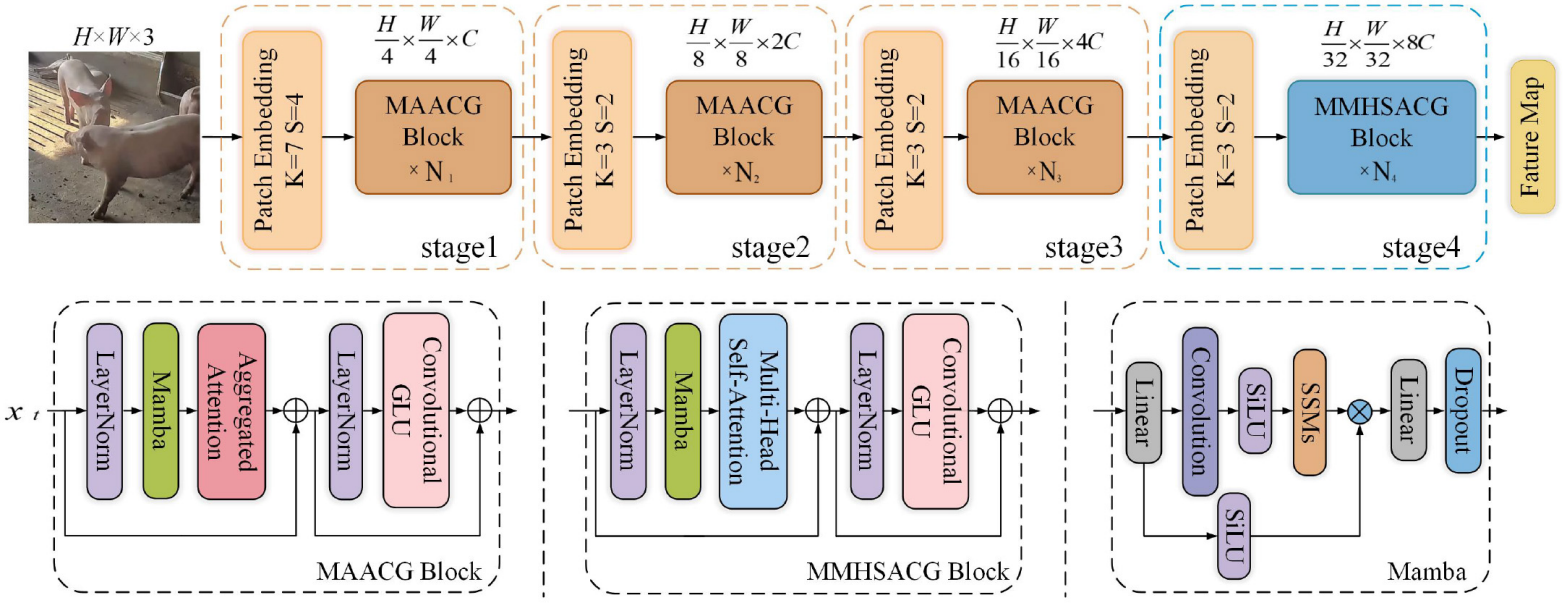
Diagram of the TransNeXtM module.

As shown in Figure 4, the TransNeXtM mainly contains a four-stage hierarchical architecture (16). Specifically, the first three stages utilize the MAACG Block, which incorporates a Mamba (17) unit, an Aggregated Attention Mechanism, and a Convolutional GLU (Convolutional Gate-controlled Linear Unit) (18), respectively. Additionally, the MMHSACG Block is employed in the final stage, which includes a Mamba unit, a Multi-Head Self-Attention Mechanism (19), and a Convolutional GLU. Notably, in the fourth stage, the small size of the feature maps makes the application of Multi-Head Self-Attention mechanism particularly suitable. This mechanism can effectively capture more abundant features without significantly increasing the computation time.

##### 2.2.1.1 Mamba

In order to enhance the discrimination of the model in pig behavior recognition task, we introduce the Mamba (17) unit into the MAACG and MMHSACG Blocks of the TransNeXtM module, as show in Figure 4, aiming to filter out irrelevant and redundant information. The Mamba unit we utilized can be formulated as follows:


(1) $$\begin{array}{c} h_{t}=\bar{A}h_{t-1}+\bar{B}x_{t} \end{array}$$

(2) $$\begin{array}{c} \bar{A}=exp\left(\Delta A\right) \end{array}$$

(3) $$\begin{array}{c} \bar{B}={\left(\Delta A\right)}^{-1}\left(exp\left(\Delta A\right)-I\right)\cdot \Delta B \end{array}$$

where *h*_t_ and *h*_t-1_ represent the state at the current and previous time steps, respectively. The parameters (Ā, $\bar{B}$) are discrete parameters transformed by (Δ, A, B), as show in Equations 2, 3. Specifically, Δ denotes sampling time-scale, *A* indicates the state transition matrix, *B* is the transition matrix for input features, and *I* stands for the identity matrix.


(4) $$\begin{array}{c} y_{t}=Ch_{t} \end{array}$$

where *y*_t_ represents the output feature, and the matrix *C* determines how the *h*_*t*_ is transformed into the *y*_*t*_.

##### 2.2.1.2 Aggregated Attention

This mechanism aims to combine local sliding window attention and global pooling attention through a dual-path design (20). It simulates the flexibility of eye movements and the capacity to process multi-scale information. Consequently, it enables the model to obtain more comprehensive features. The formulation of this mechanism is presented as follows:


(5) $$\begin{array}{c} F_{AA}\left(X_{\left(i,j\right)}\right)=\left(Z_{\left(i,j\right)\sim \rho \left(i,j\right)}+{\hat{Q}}_{\left(i,j\right)}T\right)V_{\rho \left(i,j\right)}+Z_{\left(i,j\right)\sim \sigma \left(X\right)}V_{\sigma \left(X\right)} \end{array}$$

where (*i, j*) indicates the coordinates of a center pixel, ρ(*i, j*) defines a set of pixels within a sliding window centered at (*i, j*), this representation specifically focuses on local region. On the other hand, σ(*X*) represents a feature set obtained from a whole pooled feature map, which covers the information of the global region. *Z*_(*i, j*)~ρ(*i, j*)_ and *Z*_(*i, j*)~σ(*X*)_ respectively represent attention weight matrices computed based on the sliding window and the pooled features. $\hat{Q}$ is the query matrix, *T* denotes a learnable token, and *V* stand for the value matrix.


(6) $$\begin{array}{c} Z_{\left(i,j\right)\sim \rho \left(i,j\right)},Z_{\left(i,j\right)\sim \sigma \left(X\right)}=Split\left(Z_{\left(i,j\right)}\right)\;with\;size\left[k^{2},H_{p}W_{p}\right] \end{array}$$

where *k*×*k* represents the fixed window size, and *H*_*p*_×*W*_*p*_ indicates the pooling size.


(7) $$\begin{array}{c} Z_{\left(i,j\right)}=softmax\left(\tau \log N*Concat\left(S_{\left(i,j\right)\sim p\left(i,j\right)},S_{\left(i,j\right)\sim \sigma \left(X\right)}\right)+B_{\left(i,j\right)}\right) \end{array}$$

where τ is a learnable variable initialized to $\frac{1}{0.24}$. *N* denotes the count of effective keys each query interacts with, and *Concat* stands for concatenation (21). *S*_(*i, j*)~*p*(*i, j*)_ and *S*_(*i, j*)~σ(*X*)_ reflect the relevance of local and global features, respectively. Additionally, *B* represents the bias.


(8) $$\begin{array}{c} S_{\left(i,j\right)\sim \rho \left(i,j\right)}=\left({\hat{Q}}_{\left(i,j\right)}+Q_{E}\right){\hat{K}}_{\rho \left(i,j\right)}^{T} \end{array}$$

(9) $$\begin{array}{c} S_{\left(i,j\right)\sim \sigma \left(X\right)}=\left({\hat{Q}}_{\left(i,j\right)}+Q_{E}\right){\hat{K}}_{\sigma \left(x\right)}^{T} \end{array}$$

where $\hat{Q}$ and $\hat{K}$ stand for the query matrix and the key matrix, respectively. *Q*_*E*_ denotes the learnable parameter matrix.

##### 2.2.1.3 Multi-Head Self-Attention

The Multi-Head Self-Attention mechanism maps the input sequence into multiple distinct representation heads (19). Each head then independently performs self-attention calculations on the input sequence. Consequently, this approach allows the mechanism to capture diverse levels of information within the input, thereby enhances the model's feature extraction capabilities.

The Multi-Head Self-Attention is calculated by the following equations.


(10) $$\begin{array}{c} head_{u}=Attention\left(QW_{u}^{Q},KW_{u}^{K},VW_{u}^{V}\right) \end{array}$$

(11) $$\begin{array}{c} Attention\left(Q,K,V\right)=soft\max\left(\frac{QK^{T}}{\sqrt{d_{k}}}\right)V \end{array}$$

(12) $$\begin{array}{c} MultiHead\left(Q,K,V\right)=Concat\left(head_{1},\ldots ,head_{u}\right)W^{O} \end{array}$$

where $W_{u}^{Q}$, $W_{u}^{K}$, $W_{u}^{V}$, and *W*^*O*^ denote the learnable weight matrix, *d*_*k*_ indicates the dimension of the key vector. Additionally, *u* is the number of heads.

##### 2.2.1.4 Convolutional GLU

**Figure 5:**
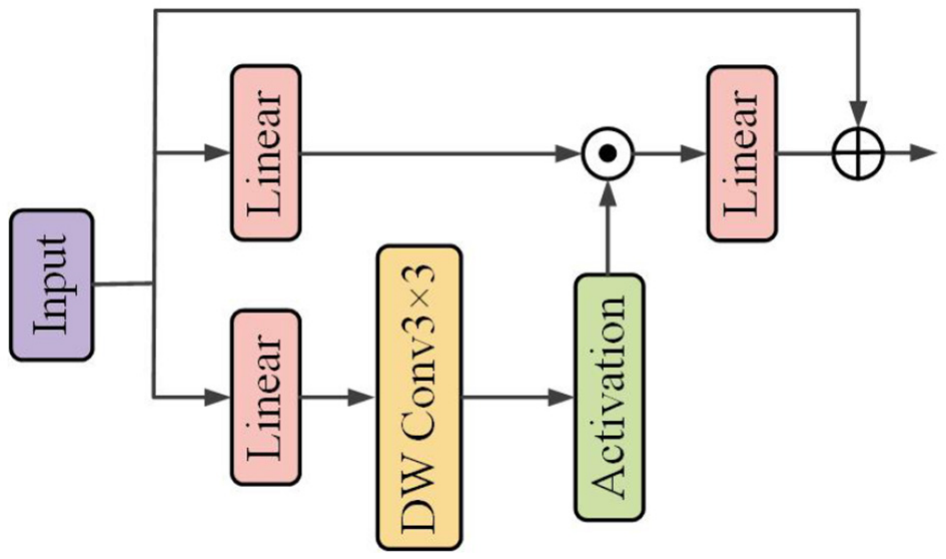
Flowchart illustrating a neural network architecture. It starts with an input, followed by two parallel linear layers. The first connects directly to an addition operation. The second goes through a depthwise convolution labeled “DW Conv 3 × 3” and an activation layer before being added to the linear layer. The final result is the output. An illustration of the Convolutional GLU.

The Convolutional GLU integrates the Gated Linear Unit (GLU) with Depthwise Convolution (DW Conv), as shown in Figure 5. It enables each token to perform channel attention computation based on the image features of its nearest neighbors, thereby enhancing the model's capability in capturing local features.

#### 2.2.2 sLSTM module

The temporal sequence module selected in this paper is sLSTM (22), as extended variant of LSTM (23). Its core improvement lies in the introduction of an exponential gated activation function (24) and a normalized state, enabling the sLSTM model to excel in capturing long temporal sequence dependencies. The specific formula is as follows:


(13) $$\begin{array}{c} c_{t}=f_{t}c_{t-1}+i_{t}z_{t} \end{array}$$

where *c*_t_ represents the state held by the cell at time step *t, f*_t_ and *i*_t_ correspond to the forget gate and the input gate, respectively.


(14) $$\begin{array}{c} z_{t}=\varphi \left({\tilde{z}}_{t}\right)\;,\;{\tilde{z}}_{t}=w_{z}^{T}x_{t}+r_{z}h_{t-1}+b_{z} \end{array}$$

where *z*_t_ denotes the unit input, which is a candidate unit state calculated based on the current input and the previous state. Here, φ represents the activation function of the unit input gate, ${\tilde{z}}_{t}$ is an intermediate variable, *w* denotes the weight matrix, *r* indicates a learnable parameter, and *b* represents the bias.


(15) $$\begin{array}{c} n_{t}=f_{t}n_{t-1}+i_{t} \end{array}$$

where *n*_t_ denotes the normalized state, which represents equivalent to adding a large denominator to prevent overflow, as the exponential activation function may produce excessively large values.


(16) $$\begin{array}{c} h_{t}=o_{t}{\tilde{h}}_{t}\;,\;{\tilde{h}}_{t}=c_{t}/n_{t} \end{array}$$

where *h*_t_ represents the hidden state and *o*_t_ denotes the output gate, while ${\tilde{h}}_{t}$ indicates an intermediate variable. The *n*_t_ is employed to prevent overflow issues.

Subsequently, we proceed to introduce the output gates, input gates, and forget gates.


(17) $$\begin{array}{c} o_{t}=\sigma \left({\tilde{o}}_{t}\right)\;,\;{\tilde{o}}_{t}=w_{o}^{T}x_{t}+r_{o}h_{t-1}+b_{o} \end{array}$$

where ${\tilde{o}}_{t}$ indicates an intermediate variable.


(18) $$\begin{array}{l} i_{t}=exp\left(\log\left(i_{t}^{\prime }\right)-m_{t}\right)\;,\\ \;i_{t}^{\prime }=exp\left({\tilde{i}}_{t}^{\prime }\right)\;,\;{\tilde{i}}_{t}^{\prime }=w_{i}^{T}x_{t}+r_{i}h_{t-1}+b_{i} \end{array}$$

where *i*_*t*_ denotes the stabilized input gate adjusted by *m*_t_. *exp* indicates the exponential function. Since the exponential function grows faster than the *sigmoid* function and is more sensitive to input changes, so that the model can capture the changes in input information faster. ${\tilde{i}}_{t}^{\prime }$ represents an intermediate variable.


(19) $$\begin{array}{c} f_{t}=exp\left(\log\left(f_{t}^{\prime }\right)+m_{t-1}-m_{t}\right)\;,\\ \;f_{t}^{\prime }=\sigma \left({\tilde{f}}_{t}^{\prime }\right)\;,\;{\tilde{f}}_{t}^{\prime }=w_{f}^{T}x_{t}+r_{f}h_{t-1}+b_{f} \end{array}$$

where *f*_*t*_ indicates the stabilized forget gate, it is also adjusted according to the value of *m*_t_. σ represents the gated activation function *sigmoid*, and ${\tilde{f}}_{t}^{\prime }$ denotes an intermediate variable.


(20) $$\begin{array}{c} m_{t}=\max\left(\log\left(\overset{\prime }{\underset{t}{f}}\right)+m_{t-1},\log\left(i_{t}^{\prime }\right)\right) \end{array}$$

where *m*_t_ denotes the stabilizer state. *log* indicates the exponential inverse operation, which is equivalent to using *log* to degrade $i_{\text{t}}^{\prime }$ and $f_{\text{t}}^{\prime }$ to avoid overflow.

### 2.3 The loss function

To effectively train the proposed model S_TransNeXtM, the common loss function for recognition tasks are employed. The detailed definition of the loss can be presented as follows:


(21) $$\begin{array}{c} Loss=-\overset{N}{\underset{n=1}{\sum }}\overset{M}{\underset{m=1}{\sum }}p_{n}^{m}\log\left({\hat{p}}_{n}^{m}\right) \end{array}$$

where *N* and *M* represent the number of samples and categories, respectively. $p_{n}^{m}$ indicates the true label for the respective sample, and ${\hat{p}}_{n}^{m}$ demonstrates the prediction label for the corresponding sample.

## 3 Experiments and analysis

A series of comprehensive experiments were conducted to evaluate the proposed model from multiple perspectives, with detailed designs and documentation systematically presented. All experimental procedures were executed on a hardware system configured with 125 GB RAM, an Intel i7-7800X CPU operating at 3.50 GHz, and an NVIDIA TITAN Xp GPU equipped with 12GB GDDR5X memory. The Ubuntu 20.04.6 LTS operating system was employed as the software foundation, while Python 3.12.4 served as the primary programming environment. Key libraries included PyTorch 2.1.2, Transformers 4.35.0, NumPy 2.0.1, and Pandas 2.2.2. Hyperparameters were systematically set as follows: batch size was configured to 4, initial learning rate was established at 0.001, and training was conducted over 200 epochs using the Adam optimizer.

In this experiment, all images were uniformly resized to 224 × 224 pixels. The input is [4, 24, 3, 224, 224], which represents batch_size, num_frames, channels, height and width respectively. The output is [4, 6], which represents batch_size and num_class, corresponding to the classification results of the pig behaviors.

### 3.1 Evaluate the effectiveness of the Mamba unit

To verify the performance of the Mamba (17) unit in S_TransNeXtM, comparisons are conducted between models that with and without the Mamba unit. Specifically, several basic models such as Swin Transformer, ConvNeXt, and TransNeXt are utilized. Comparison results are presented in Table 2, where models with the Mamba unit are written as SwinM Transformer, ConvNeXtM, and TransNeXtM, respectively.

**Table 2:** Comparison of different models with and without Mamba.

**Model**	**Accuracy (%)**	**Loss**
Swin Transformer	90.51	0.4902
**SwinM Transformer**	**92.00**	**0.4690**
ConvNeXt	91.20	0.4880
**ConvNeXtM**	**92.35**	**0.4564**
TransNeXt	92.88	0.4777
**TransNeXtM**	**93.25**	**0.4521**

The bold values indicate that the performance of this model is the best.

Table 2 demonstrates that models with Mamba unit outperform their counterparts without it. Specifically, the SwinM Transformer achieves 92.00% accuracy, making a 1.49% improvement compared to the Swin Transformer. The accuracy of the ConvNeXtM is 92.35%, surpassing the ConvNeXt by 1.15%. The TransNeXtM demonstrates the highest accuracy of 93.25%, a 0.37% increase compared to the TransNeXt. Furthermore, the loss values of the SwinM Transformer, the ConvNeXtM, and the TransNeXtM are 0.4690, 0.4564, and 0.4521, respectively, representing reductions of 0.0212, 0.0316, and 0.0259 compared to their non-Mamba counterparts.

Models with Mamba unit achieve superior accuracy in pig behavior recognition. This is attributed to Mamba's ability to filter noise and redundant information. Consequently, the model with Mamba unit is able to capture more discriminative features, leading to superior performance in the pig behavior recognition task.

### 3.2 Evaluate the effectiveness of the Aggregate Attention Mechanism

In order to assess the performance of the Aggregate Attention Mechanism in S_TransNeXtM, a comparative analysis is carried out between models with and without this mechanism. Comparison results are demonstrated in Table 3, where the model with the Aggregate Attention Mechanism is denoted as w_AggAttn(TransNeXtM) and the model without it is written as wo_AggAttn, respectively.

**Table 3:** Comparison of different models with and without the Aggregate Attention Mechanism.

**Model**	**Accuracy (%)**	**Loss**
wo_AggAttn	93.04	0.4665
**w_AggAttn**	**93.20**	**0.4521**

The bold values indicate that the performance of this model is the best.

The results in Table 3 indicates that the w_AggAttn model demonstrates superior performance compared to the wo_AggAttn model. Specifically, the accuracy of the TransNeXtM(w_AggAttn), which employed the Aggregate Attention Mechanism, reached 93.20%, marking a 0.16% improvement over the wo_AggAttn model. Furthermore, the loss value of the TransNeXtM is 0.4521, a reduction of 0.0144 compared to the wo_AggAttn model.

The model utilizing the Aggregate Attention Mechanism demonstrates superior performance. This mechanism mimics the biological visual system, effectively processes information across various scales, and enhances the model's global perception capabilities. Consequently, it enhances the model's performance in pig behavior recognition task.

### 3.3 Evaluate the effectiveness of the Convolutional GLU

This subsection focuses on validating the impact of Convolutional GLU in the S_TransNeXtM for pig behavior recognition. Specifically, we employ models both with and without the Convolutional GLU for this validation. The comparison results are presented in Table 4, where the model without the Convolutional GLU is written as wo_Convolutional GLU, and the model with the Convolutional GLU is denoted as w_Convolutional GLU(TransNeXtM). Notably, the wo_Convolutional GLU model utilizes the original MLP module.

**Table 4:** Comparison of different models with and without the Convolutional GLU.

**Model**	**Accuracy (%)**	**Loss**
wo_Convolutional GLU	92.29	0.4760
**w_Convolutional GLU**	**93.20**	**0.4521**

The bold values indicate that the performance of this model is the best.

Table 4 shows that the w_Convolutional GLU model performs better than the wo_Convolutional GLU model. Specifically, the accuracy of the TransNeXtM is 93.20%, which is 0.91% higher than that of the wo_Convolutional GLU model. The loss of the TransNeXtM is 0.4521, decreasing by 2.39% compared to the wo_Convolutional GLU model.

**Figure 6:**
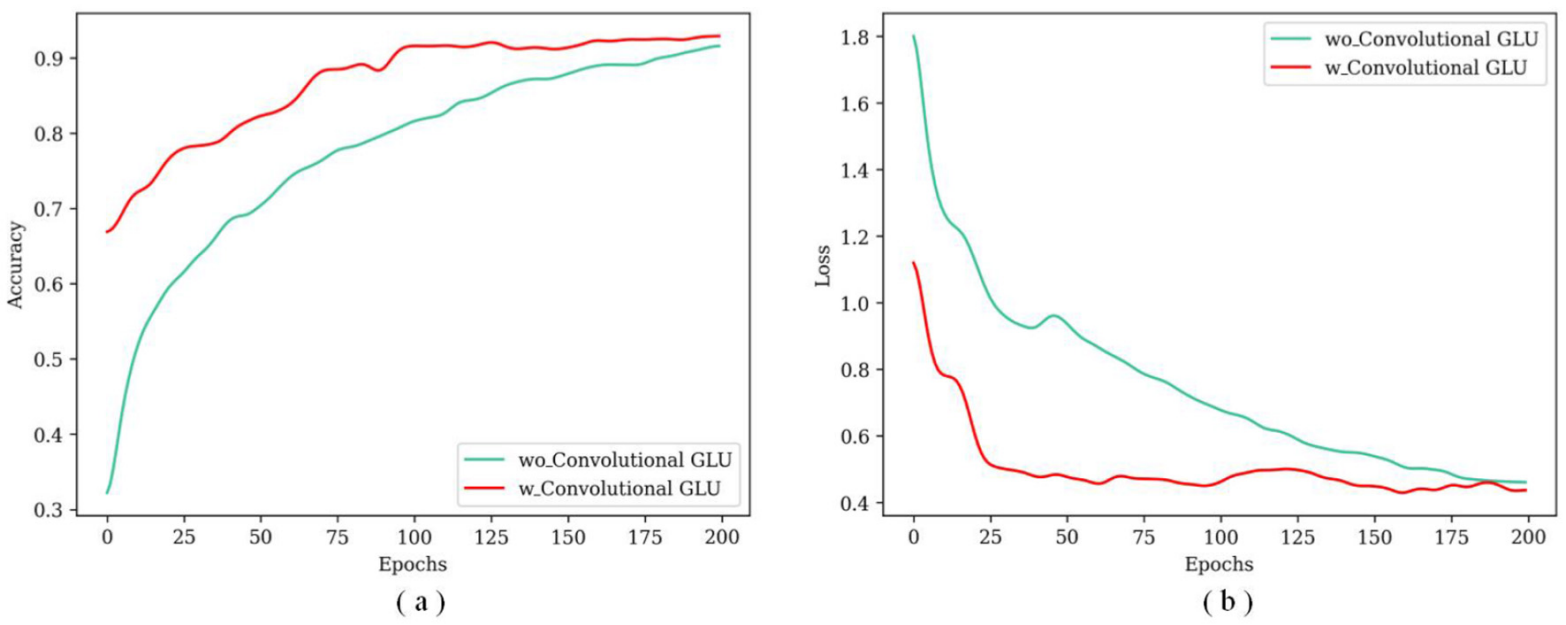
The accuracy and loss curves under different epochs for models with or without the Convolutional GLU. **(a)** Is for accuracy and **(b)** is loss.

In Figure 6a, the TransNeXtM exhibits superior accuracy compared to the model without the Convolutional GLU. Figure 6b illustrates that the loss of the TransNeXtM is lower. In conclusion, the TransNeXtM demonstrates improved performance in the task of pig behavior recognition.

The model with the Convolutional GLU outperforms the one that without, because the Convolutional GLU integrates the GLU with DW Conv. This integration enables each token to perform channel attention computation based on the image features of its nearest neighbors. Consequently, this mechanism enhances the model's ability to capture local features, thereby improving overall performance in pig behavior recognation task.

### 3.4 Evaluate the effectiveness of temporal sequence module

In this section, we design two experiments to verify the effectiveness of the temporal sequence module. The first experiment is aimed at evaluating the effectiveness of different temporal sequence modules. The second experiment focuses on comparing models with and without the temporal sequence module.

#### 3.4.1 Evaluate the effectiveness of different temporal sequence modules

To evaluate the effectiveness of different temporal sequence module, several models with diverse temporal module (GRU (18), LSTM (23) and sLSTM (22)) are utilized for comparative analysis. Specifically, these models are denoted as TransNeXtM_GRU, TransNeXtM_LSTM and S_TransNeXtM(TransNeXtM_sLSTM). The results are shown in Table 5.

**Table 5:** Comparison results of different temporal sequence modules.

**Model**	**Accuracy (%)**	**Loss**
TransNeXtM	93.25	0.4521
TransNeXtM_GRU	93.42	0.5981
TransNeXtM_LSTM	93.38	0.6579
**S_TransNeXtM**	**94.53**	**0.3350**

The bold values indicate that the performance of this model is the best.

Table 5 illustrates that the S_TransNeXtM outperforms all other models in pig behavior recognition task. Specifically, the S_TransNeXtM model reaches 94.53% accuracy, which is 1.11% and 1.55% higher than those of the TransNeXtM_GRU and the TransNeXtM_LSTM. Furthermore, its loss value is 0.3350, which is lower than those of the TransNeXtM_GRU and the TransNeXtM_LSTM.

**Figure 7:**
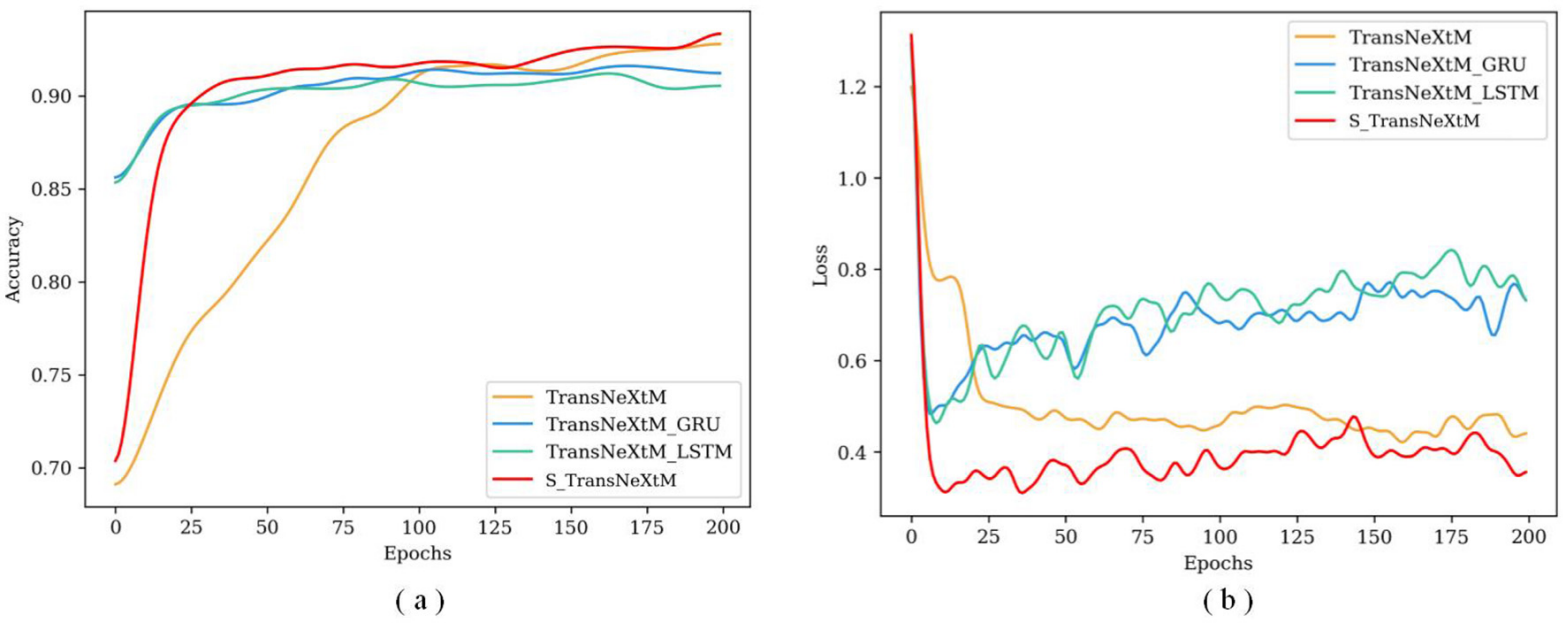
The accuracy and loss curves under different epochs for models with different temporal sequence modules. **(a)** Is for accuracy and **(b)** is loss.

To further validate the effectiveness of different temporal sequence modules, Figure 7 reports the accuracy and loss values of these models under different training epochs.

Figure 7a demonstrates that the accuracy of the S_TransNeXtM exceeds those of both the TransNeXtM_GRU and the TransNeXtM_LSTM. Additionally, Figure 7b shows that the loss of the S_TransNeXtM model during training epochs is the lowest among the three models. This further confirms the effectiveness of the sLSTM temporal sequence module.

From now on, we will default to considering the temporal sequence module mentioned as the most effective sLSTM.

#### 3.4.2 Evaluate the effectiveness when with or without the temporal sequence module

This subsection focuses on validating the impact of temporal sequence module for pig behavior recognition. Concretely, several models including the SwinM Transformer, the ConvNeXtM, and the TransNeXtM, with or without temporal sequence module, are employed for this validation. The comparison results are detailed in Table 6.

**Table 6:** Comparison results of models with or without temporal sequence module.

**Model**	**Accuracy (%)**	**Loss**
SwinM Transformer	92.00	0.4690
**S_SwinM Transformer**	**92.70**	**0.4382**
ConvNeXtM	92.35	0.4564
**S_ConvNeXtM**	**93.70**	**0.3900**
TransNeXtM	93.25	0.4521
**S TransNeXtM**	**94.53**	**0.3350**

The bold values indicate that the performance of this model is the best.

Table 6 indicates that those models with temporal sequence module perform better than those without. Specifically, the accuracy of the S_SwinM Transformer is 92.70%, exceeding the SwinM Transformer by 0.7%. The S_ConvNeXtM achieves 93.70% accuracy, outperforming 1.35% compared to the ConvNeXtM. The S_TransNeXtM achieves 94.53% recognition accuracy, representing an increase of 1.28% compared to the TransNeXtM. Furthermore, the S_SwinM Transformer demonstrates a loss value of 0.4382, which is 0.0308 lower than that of the SwinM Transformer. The loss value of the S_ConvNeXtM is 0.3900, a reduction of 0.0664 compared to the ConvNeXtM. The S_TransNeXtM's loss value is 0.4521, representing a decrease of 0.0256 compared to the TransNeXtM.

The reason why S_TransNeXtM achieves superior accuracy in analyzing pig behavior recognition is because the sLSTM with an exponential gated activation function and normalized state, which enables the model to effectively capture longer temporal sequence dependencies underlying the video. In contrast, although the GRU and the LSTM are also tools for processing temporal sequence data, they fail to reach the level of the sLSTM in terms of the depth of utilizing temporal dependency relationships, thereby affecting overall performance. Meanwhile, this unique design of sLSTM enhances the accuracy of the model's data processing capabilities, making models with the sLSTM achieve superior accuracy in pig behavior recognition.

In summary, for the task of pig behavior recognition, models with the sLSTM outperforms other models with different temporal sequence modules in terms of performance. Additionally, models with temporal sequence modules tend to exhibit better performance than without.

### 3.5 Evaluate the effectiveness of different models

To verify the effectiveness of our proposed model, S_TransNeXtM, several popular models, including ViT (Vision Transformer) (25), Swin Transformer (26), ConvNeXt (27), TransNeXt (20), EfficientViT (28), TransNeXtM and CNN-LSTM (11) are employed for comparison. The comparison results are shown in Table 7.

**Table 7:** Comparison result of S_TransNeXtM with different transformer models.

**Model**	**Accuracy (%)**	**Loss**	**Precision (%)**	**Recall (%)**	**F1-Score (%)**
ViT	83.21	0.8597	83.79	83.44	83.99
Swin Transformer	90.51	0.4902	90.88	90.32	90.40
ConvNeXt	92.52	0.3991	92.48	92.52	92.74
TransNeXt	92.88	0.4777	92.87	92.87	92.86
EfficientViT	90.51	0.4616	90.57	90.57	90.50
CNN-LSTM	90.00	0.4291	90.14	89.92	89.82
TransNeXtM	93.25	0.4521	93.25	93.21	93.20
**S_TransNeXtM**	**94.53**	**0.3350**	**94.52**	**94.54**	**94.51**

The bold values indicate that the performance of this model is the best.

Table 7 demonstrates that our proposed S_TransNeXtM model achieves the best performance across all evaluation criteria. Specifically, the S_TransNeXtM achieves a top accuracy of 94.53%, surpassing ViT (83.21%), Swin Transformer (90.51%), ConvNeXt (92.52%), TransNeXt (92.88%), EfficientViT (90.51%), CNN-LSTM (90.00%) and TransNeXtM (93.20%) by margins of 11.32%, 4.02%, 2.01%, 1.65%, 4.02%, 4.53% and 1.33% respectively. Furthermore, the S_TransNeXtM attains the lowest loss value of 0.335, showing 61.04%, 31.66%, 13.09%, 29.87%, 27.43%, 38.43% and 25.9% relative reductions compared to ViT (0.8597), Swin Transformer (0.4902), ConvNeXt (0.3991), TransNeXt (0.4777), EfficientViT (0.4616), CNN-LSTM (0.4291) and TransNeXtM (0.4521). In terms of Precision, Recall, and F1-Score, the S_TransNeXtM achieved 94.52%, 94.54%, and 94.51%, respectively, which were 1.36% to 10.37%, 1.43% to 11.1% and 1.41% to 10.61% higher than the other models.

**Figure 8:**
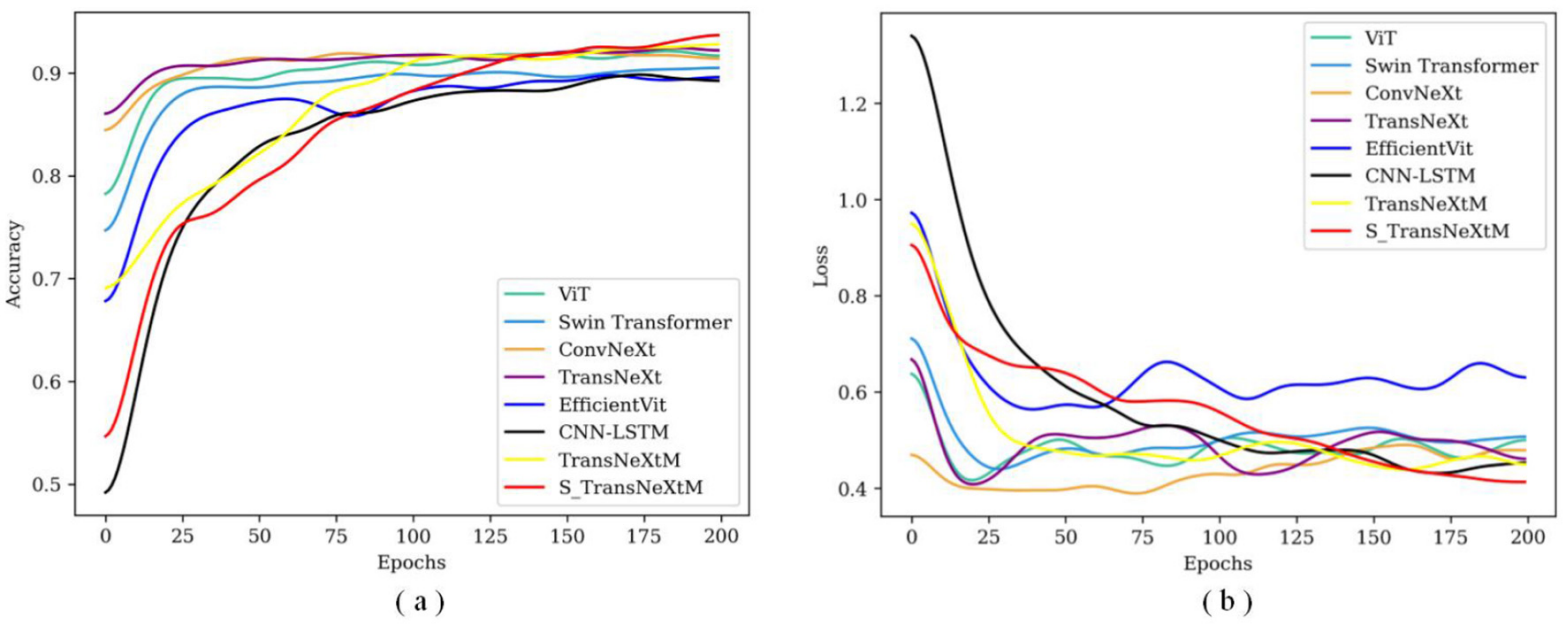
The accuracy and loss curves of S_TransNeXtM with different transformer models. **(a)** Is for accuracy and **(b)** is loss.

To further validate the effectiveness of the S_TransNeXtM, Figure 8 shows the accuracy and loss curves of the comparison models under different epochs.

In Figure 8a, the accuracy of the S_TransNeXtM surpasses that of the ViT, Swin Transformer, ConvNeXt, TransNeXt and EfficientViT models. Furthermore, in Figure 8b, the S_TransNeXtM model achieves the lowest loss. These results further validate the effectiveness of the S_TransNeXtM.

Table 8 demonstrates the superior performance of the S_TransNeXtM model in six behavioral recognition tasks for pigs. Specifically, this model achieved the highest accuracy in recognizing the three behaviors of “Eating,” “Lying,” and “Walking.” For the recognition of “Drinking,” “Fighting,” and “Exploring” behavior, although the result was slightly inferior to the other models, it still reached a suboptimal level. The accuracy gap in these three categories arises from inherent challenges. Specifically, “Drinking” involves subtle spatiotemporal patterns with minimal head movement and short duration, leading to weaker feature distinguishability. “Fighting” recognition accuracy is constrained by limited data quality, as video blur weakens the model's ability to capture key motion features of pigs. “Exploring” is difficult to distinguish from “Walking” due to their similar characteristics. Despite these category-specific limitations, the S_TransNeXtM effectively captures dominant behavioral features, ensuring robust overall performance.

**Table 8:** Comparison of the recognition performance of different models in six pig behavior categories.

**Model**	**Metric**	**Category**						**Avg Prec**	**Avg Rec**	**Avg F1**
		**Drinking**	**Eating**	**Fighting**	**Exploring**	**Lying**	**Walking**			
Vit	Precision	88.30	91.67	86.05	80.00	94.12	62.62	83.94	83.21	83.28
	Recall	97.65	90.72	85.06	65.98	85.11	76.14			
	F1-Score	92.74	91.19	85.55	72.32	89.39	68.72			
Swin Transformer	Precision	96.47	95.92	94.94	78.95	94.79	84.21	90.77	90.51	90.44
	Recall	96.47	96.91	86.21	**92.78**	96.81	72.73			
	F1-Score	96.47	96.41	90.36	85.31	95.79	78.05			
ConvNeXt	Precision	93.41	95.96	96.43	90.72	94.44	83.91	92.52	92.52	92.49
	Recall	**100.00**	97.93	93.10	90.72	90.43	82.95			
	F1-Score	96.59	96.94	94.74	90.72	92.39	83.43			
TransNeXt	Precision	96.43	97.87	88.89	90.63	95.79	87.64	92.94	92.88	92.90
	Recall	95.29	94.85	91.95	89.69	96.81	88.64			
	F1-Score	95.86	96.34	90.40	90.16	96.30	88.14			
Efficient Vit	Precision	95.45	97.89	87.64	93.10	91.49	77.89	90.73	90.51	90.55
	Recall	98.82	95.88	89.66	83.51	91.49	84.09			
	F1-Score	97.11	96.88	88.64	88.04	91.49	80.87			
CNN-LSTM	Precision	92.22	93.75	85.26	84.11	97.67	87.84	90.18	89.96	89.87
	Recall	97.65	92.78	93.10	92.78	89.36	73.86			
	F1-Score	94.86	93.26	89.01	88.24	93.33	80.25			
TransNeXtM	Precision	97.65	93.00	92.22	94.44	95.79	86.36	93.27	93.25	93.23
	Recall	97.65	95.88	**95.40**	87.63	96.81	86.36			
	F1-Score	97.65	94.42	93.79	90.91	96.30	86.36			
S_TransNeXtM	Precision	94.38	97.94	91.95	91.75	95.83	90.69	**94.11**	**94.53**	**94.29**
	Recall	98.82	**97.94**	91.95	91.75	**97.87**	**88.64**			
	F1-Score	96.55	97.94	91.95	91.75	96.84	89.65			
**Total Sample**		85	97	87	97	94	88			

The bold values indicate that the performance of this model is the best.

The superiority of the S_TransNeXtM model for pig behavior recognition can be attributed to the following reasons. Firstly, the S_TransNeXtM is with two modules: the TransNeXtM and the sLSTM. The bio-inspired Aggregate Attention Mechanism in TransNeXtM enables the model to capture global features effectively. Secondly, the Mamba unit in TransNeXtM allows the model to capture more discriminative features. Furthermore, the exponentially gated mechanism in sLSTM permits the model to establish long temporal sequence dependencies within the corresponding video. Consequently, the promising capabilities of global feature extraction and the establishment of long temporal sequence dependencies enhance the performance of pig behavior recognition.

### 3.6 Ablation experiments

In this section, a series of ablation experiments are conducted to systematically assess the effectiveness of the Mamba and sLSTM modules. Specifically, separate introduction of the Aggregation Attention Mechanism or Convolutional GLU module leads to a drawback. The model fails to simultaneously and effectively capture global and local image features. Consequently, the performance of the model declines markedly in the task of pig behavior recognition. Therefore, this experiment adopts the model introducing both of these two modules as the baseline (Index 1).

Based on Table 9, when both Mamba and the Aggregated Attention Mechanism are introduced (Index 2), shows no performance gain over baseline. While Mamba excels at capturing global information from sequential data, it struggles with extracting local features. Similarly, the Aggregated Attention Mechanism emphasizes global features. Due to the lack of the ability to precisely capture local features, consequently impacts performance of the model in pig behavior recognition tasks.

**Table 9:** Performance comparison of the models with different module combinations.

**Index**	**Block**			**sLSTM**	**Accuracy (%)**	**Loss**
	**Mamba**	**Aggregate Attention**	**Convolutional GLU**			
1	×	✓	✓	×	92.88	0.4777
2	✓	✓	×	×	92.29	0.4760
3	✓	×	✓	×	93.04	0.4665
4	✓	✓	✓	×	93.20	0.4521
5	×	✓	✓	✓	93.98	0.3860
6	✓	✓	✓	✓	**94.53**	**0.3500**

The bold values indicate that the performance of this model is the best.

The combination of the Mamba and Convolutional GLU modules (Index 3), it demonstrates a 0.16% increase in accuracy, and a 2.34% decrease in loss. These two modules are respectively adept at capturing global and local features, and the Mamba is more capable than the Aggregation Attention Mechanism in handling long temporal sequence dependencies. Therefore, compared with the baseline, the performance of the model has been improved.

When the Mamba unit is added (Index 4) in baseline, it exhibits a 0.32% increase in accuracy and a 5.36% decrease in loss compared to the baseline (Index 1). Furthermore, the introduction of Mamba facilitates the filtration of noise and redundant information, enabling the model to extract more discriminative pig behavioral features, which in turn improves the model's performance.

When the sLSTM modules is introduced (Index 5), it demonstrates a 1.1% increase in accuracy, a 19.2% decrease in loss. Due to the unique design of sLSTM featuring exponential gating activation functions and normalized state, the model can effectively capture longer temporal sequence dependencies in videos, improve the accuracy of its data processing, and thus achieve higher accuracy in pig behavior recognition.

Finally, when the Mamba and sLSTM are simultaneously added (Index 6), the S_TransNeXtM achieves peak performance with a 1.65% increase in accuracy, a 26.7% decrease in loss compared to the baseline. This combination maximizes the utilization of their complementary strengths. Specifically, the Mamba suppresses noise and effectively captures the characteristics of pigs. Additionally, sLSTM handles long temporal sequence dependence.

By leveraging the synergistic effects among these modules, the S_TransNeXtM model addresses the limitations of traditional pig behavior recognition models in effectively extracting image features and analyzing long temporal sequence dependencies.

## 4 Conclusions

This paper proposes a novel pig behavior recognition model, S_TransNeXtM, which leverages both spatial and temporal information underlying the video. Specifically, for the spatial domain, the TransNeXtM is initially introduced, leveraging a Aggregated Attention Mechanism, a Convolutional GLU, and a Mamba unit to capture more discriminative global and local features. This allows the model to perceive more subtle differences in the pig's behavior. Additionally, in the temporal domain, sLSTM's exponential gating and stabilized states provide improved capability for processing long temporal sequence dependencies. Consequently, the S_TransNeXtM enhances the performance of pig behavior recognition. We conducted numerous experiments to validate the effectiveness of the proposed model. Experimental validation demonstrates the state-of-the-art performance with 94.53% accuracy, surpassing the mentioned methods by 0.55%–11.32% and reducing loss by 52.47%.

In the future work, we will focus on processing longer temporal sequence data to further improve the performance of pig behavior recognition, via optimizing the architecture of the model. Additionally, we will work on developing an adaptive learning mechanism that enables the model to automatically adjust its parameters. Thus, it can cope with the diversity and dynamic changes of pig behavior under different environmental conditions. Furthermore, we will invest more resources into collecting high-quality, diverse, and representative datasets of pig behavior under various conditions.

## Data Availability

The raw data supporting the conclusions of this article will be made available by the authors, without undue reservation.
